# (*E*)-3-(2,3,4,5,6-Penta­fluoro­styr­yl)thio­phene

**DOI:** 10.1107/S1600536810009992

**Published:** 2010-03-24

**Authors:** Sébastien Clément, Olivier Coulembier, Franck Meyer, Matthias Zeller, Christophe M. L. Vande Velde

**Affiliations:** a Laboratoire de Chimie des Polymères, CP206/1 Université Libre de Bruxelles, Boulevard du Triomphe, Faculté des Sciences, 1050 Bruxelles, Belgium; b Laboratory of Polymeric and Composite Materials, Center of Innovation and Research in Materials and Polymers (CIRMAP), University of Mons UMONS, Place du Parc 23, 7000 Mons, Belgium; c Department of Chemistry, Youngstown State University, One University Plaza, Youngstown, OH 44555-3663, USA; d Department of Applied Engineering, Karel de Grote University College, Salesianenlaan 30, 2660 Antwerp, Belgium

## Abstract

The reaction of thio­phene-3-carboxaldehyde and perfluoro­benzyl­triphenyl­phospho­nium bromide in the presence of sodium hydride gave the title compound, C_12_H_5_F_5_S, in 70% yield. The thiophene and perfluorophenyl groups form a dihedral angle of 5.4 (2)°. The structure is characterized by a head-to-tail organization in a columnar arrangement due to π–π inter­actions between the thio­phene and penta­fluoro­phenyl rings with centroid–centroid distances in the range 3.698 (2)–3.802 (2) Å.

## Related literature

For electronic materials with high conductivity due to complementary groups, see: Yamamoto *et al.* (2009 ▶); Hoeben *et al.* (2005 ▶). For a bottom-up approach to rational design of electronic materials, see: Lu & Lieber (2007 ▶). For thio­phene derivatives used in solar cells or oLEDs, see: Osaka & McCullough (2008 ▶); Mishra *et al.* (2009 ▶). For the structure of 2,5-dibromo-3-(2,3,4,5,6-penta­fluoro­styr­yl)thio­phene, see: Clément *et al.* (2010 ▶).
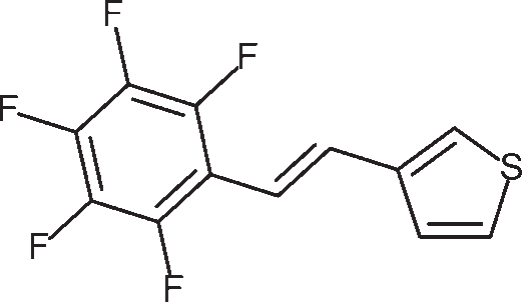


## Experimental

### 

#### Crystal data

C_12_H_5_F_5_S
*M* *_r_* = 276.22
Monoclinic, 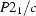

*a* = 5.8097 (15) Å
*b* = 24.581 (6) Å
*c* = 7.3224 (18) Å
β = 94.953 (4)°
*V* = 1041.8 (4) Å^3^
*Z* = 4
Mo *K*α radiation
μ = 0.36 mm^−1^
*T* = 100 K
0.31 × 0.21 × 0.05 mm

#### Data collection

Bruker SMART APEX area-detector diffractometer
Absorption correction: multi-scan (*SADABS*; Bruker, 2008 ▶) *T*_min_ = 0.637, *T*_max_ = 0.746
5781 measured reflections
3056 independent reflections
2513 reflections with *I* > 2σ(*I*)
*R*_int_ = 0.031

#### Refinement

*R*[*F* ^2^ > 2σ(*F* ^2^)] = 0.086
*wR*(*F* ^2^) = 0.186
*S* = 1.21
3056 reflections
163 parameters
H-atom parameters constrained
Δρ_max_ = 0.73 e Å^−3^
Δρ_min_ = −0.58 e Å^−3^

### 

Data collection: *APEX2* (Bruker, 2008 ▶); cell refinement: *SAINT* (Bruker, 2007 ▶); data reduction: *SAINT*; program(s) used to solve structure: *SHELXS97* (Sheldrick, 2008 ▶); program(s) used to refine structure: *SHELXL97* (Sheldrick, 2008 ▶); molecular graphics: *ORTEP-3* (Farrugia, 1997 ▶) and *Mercury* (Macrae *et al.*, 2008 ▶); software used to prepare material for publication: *WinGX* (Farrugia, 1999 ▶) and *PLATON* (Spek, 2009 ▶).

## Supplementary Material

Crystal structure: contains datablocks I, global. DOI: 10.1107/S1600536810009992/sj2740sup1.cif

Structure factors: contains datablocks I. DOI: 10.1107/S1600536810009992/sj2740Isup2.hkl

Additional supplementary materials:  crystallographic information; 3D view; checkCIF report

## Figures and Tables

**Fig. 1.**
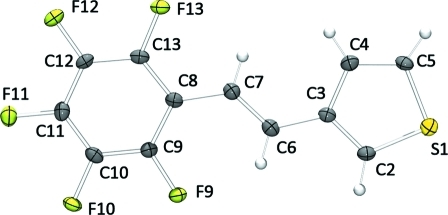
The structure of 1 with displacement ellipsoids drawn at the 50% probablity level.

**Fig. 2.**
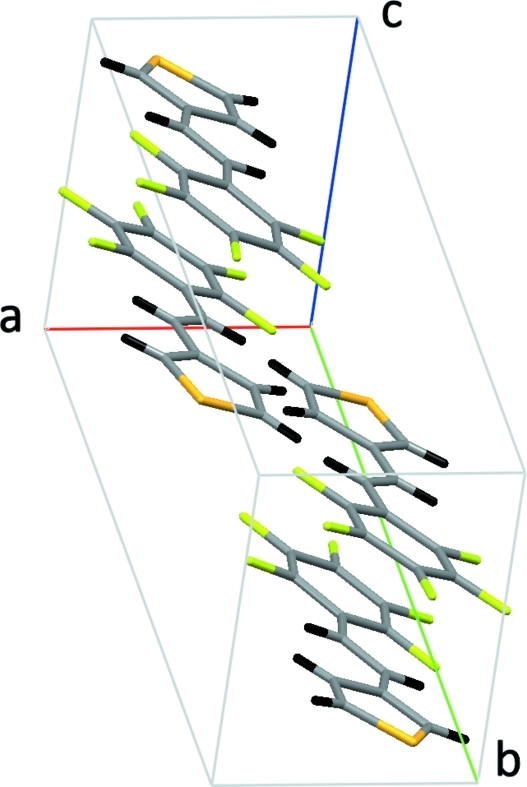
A view of the packing of 1.

## supplementary crystallographic information

### Comment

The development of new electronic devices is currently performed through the engineering of organic electronic materials composed of π-conjugated polymers. The incorporation of unsaturated systems with complementary groups takes advantage of high electronic conductivity supplemented by a supramolecular organization at the nanoscale (Yamamoto *et al.*, 2009, Hoeben *et al.*, 2005). Therefore, the rational design of new building blocks has arisen as an essential pathway to fulfill the bottom-up approach (Lu & Lieber, 2007). As a preliminary milestone, we report the structure of (*E*)-3-(perfluorostyryl)thiophene (1), an intermediate aiming at the preparation of polythiophenes with self-complementary groups. These thiophene derivatives could find applications in electronic devices with solar cell or organic light emitting diode (oLED) properties (Osaka & McCullough, 2008; Mishra *et al.*, 2009). The structure of 1 is shown in Figure 1.

(*E*)-3-(perfluorostyryl)thiophene crystallizes in the space group P2_1_/c and exhibits an almost planar molecular geometry - a slight rotation of 5.4 (2)° between the L.S. planes of the thiophene and perfluorophenyl groups is observed. The π-π stacking between the aromatic rings arranges the unsaturated compound in alternating orientations within one column due to opposite dipole moments. The distance between the thiophene-perfluorophenyl centres for successive pairs is in the range 3.698 (2)-3.802 (2) Å.

The orientation of the double bonds of successive molecules in the columns is perpendicular, in contrast with 2,5-dibromo-3-(perfluorostyryl)thiophene (Clément *et al.*, 2010), where they are parallel, due to a different arrangement of the molecules with regard to the symmetry elements in the cell, although the space group is identical.

Neighboring columns in 1 are closely packed, with the molecules in neigboring columns shifted up or down by approximately half the intermolecular distance. Between columns, there are also short S—S contacts and 2 F—F interactions. For a list of short contacts, see the "Geometric parameters" table.

### Experimental

Perfluorobenzyltriphenylphosphonium bromide (800 mg,1.53 mmol) and sodium hydride (80 mg, 2 mmol) are stirred in 5 ml of DMF during15 min. Then, thiophenecarboxaldehyde (0.13 ml, 1.53 mmol) is added and the mixture is heated at 50 °C. After 16 h, the reaction is hydrolyzed and the solid residue is filtered off. The compound is purified by chromatography on silica gel with hexane/CH_2_Cl_2_ (4:1) to give (*E*)-3-(perfluorostyryl)thiophene in 70% yield. Crystals of 1 were obtained by slow evaporation of a saturated dichloromethane solution. ^1^H NMR (300 MHz, CDCl_3_):d7.43 (d, 1 H, CH=, ^3^*J*_H—H_= 16.5, vinyl-*H*), 7.37 (m, 3 H,3 H_ar_), 6.82 (d, 1 H, CH=, ^3^*J*_H—H_ = 16.5, vinyl-*H*);^13^C{^1^H} NMR (CDCl_3_): d 145.7(C-8, C-12), 142.3 (C-10), 138.8 (C-9, C-11), 130.5, 126.1, 124.3, 123.8 (C-1, C-2, C-3, C-4, C-5, C-6); 111.8 (C-7); ^19^F NMR (CDCl_3_): d -140.9 (2 F, F*_ortho_*), -154.4 (1 F, F*_para_*), -162.9 (2 F, F*_meta_*); ESI-MS (m/z): 276(100, M+), 257 (92, M+ -F).

### Refinement

All H-atoms were positioned geometrically and refined using a riding model with d(C-H) = 0.93 Å,  U_iso_ = 1.2U_eq_ (C).

### Crystal data

**Table:**

C_12_H_5_F_5_S	*F*(000) = 552
*M**_r_* = 276.22	*D*_x_ = 1.761 Mg m^−^^3^
Monoclinic, *P*2_1_/*c*	Mo *K*α radiation, λ = 0.71073 Å
Hall symbol: -P 2ybc	Cell parameters from 2814 reflections
*a* = 5.8097 (15) Å	θ = 2.9–31.1°
*b* = 24.581 (6) Å	µ = 0.36 mm^−^^1^
*c* = 7.3224 (18) Å	*T* = 100 K
β = 94.953 (4)°	Plate, colourless
*V* = 1041.8 (4) Å^3^	0.31 × 0.21 × 0.05 mm
*Z* = 4	

### Data collection

**Table:**

Bruker SMART APEX area-detector diffractometer	3056 independent reflections
Radiation source: fine-focus sealed tube	2513 reflections with *I* > 2σ(*I*)
graphite	*R*_int_ = 0.031
ω scans	θ_max_ = 31.2°, θ_min_ = 1.7°
Absorption correction: multi-scan (*SADABS*; Bruker, 2008)	*h* = −5→8
*T*_min_ = 0.637, *T*_max_ = 0.746	*k* = −25→35
5781 measured reflections	*l* = −10→8

### Refinement

**Table:**

Refinement on *F*^2^	Primary atom site location: structure-invariant direct methods
Least-squares matrix: full	Secondary atom site location: difference Fourier map
*R*[*F*^2^ > 2σ(*F*^2^)] = 0.086	Hydrogen site location: inferred from neighbouring sites
*wR*(*F*^2^) = 0.186	H-atom parameters constrained
*S* = 1.21	*w* = 1/[σ^2^(*F*_o_^2^) + (0.0193*P*)^2^ + 5.9694*P*] where *P* = (*F*_o_^2^ + 2*F*_c_^2^)/3
3056 reflections	(Δ/σ)_max_ < 0.001
163 parameters	Δρ_max_ = 0.73 e Å^−^^3^
0 restraints	Δρ_min_ = −0.58 e Å^−^^3^
3 constraints	

### Special details

**Table:**

Geometry. All esds (except the esd in the dihedral angle between two l.s. planes) are estimated using the full covariance matrix. The cell esds are taken into account individually in the estimation of esds in distances, angles and torsion angles; correlations between esds in cell parameters are only used when they are defined by crystal symmetry. An approximate (isotropic) treatment of cell esds is used for estimating esds involving l.s. planes.
Refinement. Refinement of *F*^2^ against ALL reflections. The weighted *R*-factor wR and goodness of fit *S* are based on *F*^2^, conventional *R*-factors *R* are based on *F*, with *F* set to zero for negative *F*^2^. The threshold expression of *F*^2^ > σ(*F*^2^) is used only for calculating *R*-factors(gt) etc. and is not relevant to the choice of reflections for refinement. *R*-factors based on *F*^2^ are statistically about twice as large as those based on *F*, and *R*- factors based on ALL data will be even larger.

### Fractional atomic coordinates and isotropic or equivalent isotropic displacement parameters (Å^2^)

**Table:**

	*x*	*y*	*z*	*U*_iso_*/*U*_eq_	
S1	0.22542 (18)	0.54717 (4)	0.56342 (15)	0.0219 (2)	
F9	−0.0433 (4)	0.80297 (10)	0.4285 (3)	0.0200 (5)	
F10	−0.0888 (4)	0.91035 (10)	0.4229 (3)	0.0211 (5)	
F12	0.6511 (4)	0.93050 (10)	0.7310 (3)	0.0218 (5)	
F13	0.6987 (4)	0.82233 (10)	0.7475 (3)	0.0205 (5)	
F11	0.2579 (5)	0.97589 (10)	0.5694 (4)	0.0244 (5)	
C4	0.4895 (7)	0.62804 (17)	0.6462 (5)	0.0181 (7)	
H4	0.6168	0.6489	0.6883	0.022*	
C13	0.5023 (6)	0.84305 (17)	0.6626 (5)	0.0160 (7)	
C3	0.2770 (6)	0.65098 (16)	0.5703 (5)	0.0154 (7)	
C8	0.3328 (6)	0.80732 (16)	0.5885 (5)	0.0154 (7)	
C12	0.4805 (6)	0.89877 (16)	0.6571 (5)	0.0162 (7)	
C11	0.2808 (7)	0.92176 (16)	0.5756 (5)	0.0188 (8)	
C9	0.1336 (7)	0.83262 (16)	0.5062 (5)	0.0161 (7)	
C6	0.2239 (7)	0.70861 (16)	0.5483 (5)	0.0183 (7)	
H6	0.0793	0.7182	0.4934	0.022*	
C10	0.1082 (7)	0.88846 (17)	0.5015 (5)	0.0185 (8)	
C7	0.3708 (7)	0.74870 (16)	0.6022 (5)	0.0178 (7)	
H7	0.5149	0.7378	0.6548	0.021*	
C2	0.1186 (7)	0.61079 (17)	0.5195 (5)	0.0179 (7)	
H2	−0.0301	0.6177	0.4670	0.022*	
C5	0.4880 (7)	0.57227 (17)	0.6512 (5)	0.0177 (7)	
H5	0.6123	0.5509	0.6961	0.021*	

### Atomic displacement parameters (Å^2^)

**Table:**

	*U*^11^	*U*^22^	*U*^33^	*U*^12^	*U*^13^	*U*^23^
S1	0.0228 (5)	0.0195 (5)	0.0229 (5)	−0.0009 (4)	−0.0003 (4)	−0.0008 (4)
F9	0.0164 (11)	0.0213 (12)	0.0216 (12)	−0.0008 (9)	−0.0019 (9)	−0.0022 (9)
F10	0.0163 (11)	0.0242 (13)	0.0219 (12)	0.0053 (9)	−0.0039 (9)	0.0004 (10)
F12	0.0203 (11)	0.0239 (13)	0.0210 (12)	−0.0070 (10)	−0.0004 (9)	−0.0039 (10)
F13	0.0150 (10)	0.0257 (13)	0.0199 (12)	0.0008 (9)	−0.0033 (9)	−0.0005 (9)
F11	0.0309 (13)	0.0179 (12)	0.0240 (13)	0.0007 (10)	0.0003 (10)	−0.0014 (10)
C4	0.0169 (16)	0.0215 (19)	0.0159 (16)	−0.0013 (14)	0.0018 (13)	−0.0014 (15)
C13	0.0131 (15)	0.024 (2)	0.0107 (15)	0.0005 (14)	0.0010 (12)	0.0007 (14)
C3	0.0158 (16)	0.0196 (19)	0.0107 (15)	0.0011 (13)	0.0010 (13)	−0.0002 (13)
C8	0.0136 (16)	0.0198 (19)	0.0133 (16)	0.0009 (13)	0.0035 (13)	0.0000 (14)
C12	0.0150 (16)	0.0206 (19)	0.0129 (16)	−0.0048 (14)	0.0013 (13)	−0.0023 (14)
C11	0.026 (2)	0.0166 (19)	0.0137 (17)	0.0001 (15)	0.0035 (15)	−0.0014 (14)
C9	0.0158 (16)	0.0185 (19)	0.0141 (16)	−0.0014 (14)	0.0013 (13)	0.0011 (14)
C6	0.0206 (17)	0.0199 (19)	0.0148 (17)	0.0032 (15)	0.0028 (13)	0.0011 (14)
C10	0.0163 (17)	0.023 (2)	0.0170 (17)	0.0047 (14)	0.0036 (14)	0.0018 (15)
C7	0.0201 (17)	0.0189 (19)	0.0144 (16)	0.0018 (14)	0.0010 (14)	−0.0002 (14)
C2	0.0156 (17)	0.022 (2)	0.0152 (16)	−0.0009 (14)	−0.0018 (13)	−0.0003 (14)
C5	0.0170 (17)	0.022 (2)	0.0133 (16)	0.0035 (14)	−0.0020 (13)	−0.0002 (14)

### Geometric parameters (Å, °)

**Table:**

S1—C2	1.703 (4)	C3—C2	1.380 (5)
S1—C5	1.718 (4)	C3—C6	1.456 (5)
F9—C9	1.345 (4)	C8—C9	1.403 (5)
F10—C10	1.348 (4)	C8—C7	1.460 (5)
F12—C12	1.338 (4)	C12—C11	1.379 (6)
F13—C13	1.350 (4)	C11—C10	1.370 (6)
F11—C11	1.338 (5)	C9—C10	1.381 (6)
C4—C5	1.371 (6)	C6—C7	1.341 (6)
C4—C3	1.425 (5)	C6—H6	0.9300
C4—H4	0.9300	C7—H7	0.9300
C13—C12	1.376 (6)	C2—H2	0.9300
C13—C8	1.394 (5)	C5—H5	0.9300
			
S1···S1^i^	3.5611 (17)	F10···H5^iii^	2.49
F9···F13^ii^	2.921 (3)	H5···F11^iv^	2.59
F10···F12^ii^	2.865 (3)	H2···F13^iii^	2.61
F10···C12^ii^	3.166 (4)	C3···C9^v^	3.392 (5)
F10···C5^iii^	3.056 (5)	C3···C13^vi^	3.365 (5)
			
C2—S1—C5	92.19 (19)	F9—C9—C10	116.9 (3)
C5—C4—C3	113.5 (4)	F9—C9—C8	120.9 (3)
C5—C4—H4	123.3	C10—C9—C8	122.3 (4)
C3—C4—H4	123.3	C7—C6—C3	124.0 (4)
F13—C13—C12	117.4 (3)	C7—C6—H6	118.0
F13—C13—C8	118.8 (4)	C3—C6—H6	118.0
C12—C13—C8	123.7 (4)	F10—C10—C11	119.8 (4)
C2—C3—C4	110.9 (4)	F10—C10—C9	119.5 (4)
C2—C3—C6	122.5 (4)	C11—C10—C9	120.7 (4)
C4—C3—C6	126.6 (4)	C6—C7—C8	128.1 (4)
C13—C8—C9	114.6 (4)	C6—C7—H7	116.0
C13—C8—C7	119.8 (3)	C8—C7—H7	116.0
C9—C8—C7	125.5 (4)	C3—C2—S1	112.5 (3)
F12—C12—C13	120.3 (3)	C3—C2—H2	123.7
F12—C12—C11	120.2 (4)	S1—C2—H2	123.7
C13—C12—C11	119.5 (4)	C4—C5—S1	110.9 (3)
F11—C11—C10	120.9 (4)	C4—C5—H5	124.6
F11—C11—C12	120.0 (4)	S1—C5—H5	124.6
C10—C11—C12	119.1 (4)		
			
C5—C4—C3—C2	0.0 (5)	C2—C3—C6—C7	177.5 (4)
C5—C4—C3—C6	179.3 (4)	C4—C3—C6—C7	−1.7 (6)
F13—C13—C8—C9	178.9 (3)	F11—C11—C10—F10	−0.7 (6)
C12—C13—C8—C9	−0.1 (6)	C12—C11—C10—F10	179.4 (3)
F13—C13—C8—C7	−0.6 (5)	F11—C11—C10—C9	179.2 (4)
C12—C13—C8—C7	−179.6 (4)	C12—C11—C10—C9	−0.6 (6)
F13—C13—C12—F12	1.1 (5)	F9—C9—C10—F10	0.7 (5)
C8—C13—C12—F12	−179.9 (3)	C8—C9—C10—F10	−179.0 (3)
F13—C13—C12—C11	−178.5 (3)	F9—C9—C10—C11	−179.3 (3)
C8—C13—C12—C11	0.5 (6)	C8—C9—C10—C11	1.1 (6)
F12—C12—C11—F11	0.4 (6)	C3—C6—C7—C8	−179.1 (4)
C13—C12—C11—F11	−180.0 (4)	C13—C8—C7—C6	176.1 (4)
F12—C12—C11—C10	−179.7 (4)	C9—C8—C7—C6	−3.3 (7)
C13—C12—C11—C10	−0.1 (6)	C4—C3—C2—S1	0.1 (4)
C13—C8—C9—F9	179.7 (3)	C6—C3—C2—S1	−179.2 (3)
C7—C8—C9—F9	−0.9 (6)	C5—S1—C2—C3	−0.2 (3)
C13—C8—C9—C10	−0.7 (6)	C3—C4—C5—S1	−0.2 (4)
C7—C8—C9—C10	178.8 (4)	C2—S1—C5—C4	0.2 (3)

Symmetry codes: (i) −*x*, −*y*+1, −*z*+1; (ii) *x*−1, *y*, *z*; (iii) *x*−1, −*y*+3/2, *z*−1/2; (iv) −*x*+1, *y*−1/2, −*z*+3/2; (v) *x*, −*y*+3/2, *z*+1/2; (vi) *x*, −*y*+3/2, *z*−1/2.
